#  The Study of Anti-Inflammatory Activity of Oil-Based Dill *(Anethum graveolens L.)* Extract Used Topically in Formalin-Induced Inflammation Male Rat Paw 

**Published:** 2012

**Authors:** Mohsen Naseri, Faraz Mojab, Mahmood Khodadoost, Mohammad Kamalinejad, Ali Davati, Rasol Choopani, Abbas Hasheminejad, Zahra Bararpoor, Shamsa Shariatpanahi, Majid Emtiazy

**Affiliations:** a *Traditional Medicine Clinical Trial Research Center, Shahed University, Tehran, Iran. *; b *School of Pharmacy, Shahid Beheshti University of Medical Sciences, Tehran, Iran. *; c *School of Medicine Shahed University, Tehran, Iran. *; d *School of Traditional Medicine, Shahid Beheshti University of Medical Sciences, Tehran, Iran.*; e *School of Iranian Traditional Medicine, Shahid Sadoughi University of Medical Sciences, Ardakan, Yazd, Iran. *

**Keywords:** Anti-inflammatory, Dill oil, Formalin test, Iranian traditional medicine

## Abstract

Inflammation is one of the symptoms of many common and harmful diseases. As it is incurable through chemical drugs, the study on this ailment using new methods and drugs seems necessary. In addition, the adverse effects of the present anti-inflammatory drugs like NSAIDS and Glucocorticoid appeared in the long time use make such study more demanded. Accordingly, in this study we examined the effects of aerial organs’ extract and seed of a plant commonly used in Iranian traditional medicine named Dill on the inflammation caused by plantar injection of formalin in rats and compared them with Diclofenac-gel.

One of the methods used for the inflammation assessment is injecting formalin in the rat paw and then measuring the paw volume by the new plethysmometer (weighing method). The assessment is done at a specific time on day for 8 days and then recorded.

This study includes 3 groups of 6 male rats: Formalin, Dill-Oil and Diclofenac-gel groups.

The Dill-Oil group received 2 g of Dill-Oil, containing 100 mg Dill-extract and the Diclofenac group received 2 g gel containing, 20 mg Diclofenac Na.

Data were analyzed with SPSS 17 using ANOVA, Kruskal-Wallis, and Repeated-Measures.

The average paw volumes changes in these groups after Formalin-induced inflammation on 1st day, were 0.31 (standard error (SEM) = 0.02), 0.30 (SEM = 0.01) and 0.32 (SEM = 0.05) respectively, with no significant difference.

Regarding the peak of inflammation on the 2nd day, it was indicated that the average inflammations in Formalin, Dill-Oil and Diclofenac-gel groups were 0.44 (SEM = 0.03), 0.15 (SEM = 0.04) and 0.36 (SEM = 0.08), respectively.

The paw volume changes in groups receiving Dill-oil and Diclofenac-gel, after the daily formalin injection in 8 days compared to the blank group, had a significant decrease (p < 0.001). The Dill group showed even more decrease in the paw volume compared to the Diclofenac one.

The results of paw volume measurement analyzed by the Plethysmometer manifest that the Dill-Oil is able to decrease the paw volume significantly.

## Introduction

Though inflammation has the ability to act as an anti-septic agent, it can also improve the healing process of the wound, together with the whole healing process, have a significant potential to cause afflictions and problems (1).

Among the most significant health problems all around the world are the acute and chronic inflammatory diseases. Although numerous medications are known to treat the inflammatory diseases, their prolonged use mostly leads to gastrointestinal intolerance, bone marrow depression, water and salt retention and *etc. *(2).

Now, day-by-day, several different synthetic drugs of better efficacy are being introduced. But apart from being effective, most of them induce adverse side effects; especially in the patients with chronic inflammatory diseases (3). Because of this, most of the research is moving towards integrating complementary medicine with the mainstream medicine to increase efficacy and to decrease side effects and costs. So in this study, «Dill», a native plant of Iran with a long history of consumption, less side effects and cost-effectiveness, is used (4).

Dill is a plant from the Apiaceae family characterized by a hollow, straight, branched off 40-120 cm long stem with aromatic odor and is cultivated in different regions of Iran. Dill, as a flavoring and nutritious plant, is used in some common Iranian foods. Dill is used as a remedy for indigestion and flatulence and is also reported to be milk secretion stimulant and cholesterol-lowering agent. Moreover, it is used as an anti-convulsion, anti-emetic, anti-cramp (in children) remedy and also recommended topically as a wound healer. On the other hand, Dill seed augments the appetite and strengthen the stomach (5).

Based on Iranian Traditional Medicine texts and definition of diseases in traditional medicine and modern medicine, «Dill» is one of the most well-known herbs that have been used in several different disorders as anti-inflammatory, diuretic, galactogogue and anti-spasmodic. In addition, Dill has consumed as stomach, liver, kidney and bladder tonic. Dill can strengthen the brain in poultice form. It can also be used as preservative, carminative and anti-infection (6-13). Experimental studies have shown that Dill is anti-bacterial (14), anti-oxidant (15), anti-bloating (16), galactogogue, diuretic, appetizer (17-18), intestinal anti-spasmodic (18-20) and also effective in curing urinary and brain disorders.

Moreover, it is effective in stomach ulcer treatment, gastric secretion inhibition and consequently stomach ulcer inhibition in rats, which seems to be due to the presence of the terpenes and flavonoids in its extract.

The major part (90%) of the Dill fruit’s oil consists of d-carvone, d-limonene, and *α*-phellandrene. The remained includes: Dillanoside, kaempferol and 3-glucuronide compound, vicenin, myristicin and other flavonoids, phenolic acids, proteins and fats (5, 23-25).

A study revealed that the hydroalcoholic extract of the Dill seed causes significantly a decrease in the inflammation and pain of the male rat (25).

According to the mentioned descriptions, we designed this study to evaluate the analgesic and anti-inflammatory effects of the aqueous extract of this plant (Dill) in sesame oil on male rats.

## Experimental

*The Dill extract preparation*

Fresh aerial parts of the Dill herb (leaves and seeds) were prepared from the farms of Shahr-e-Ray on June, 2010. The plant was scientifically identified and recorded in the School of Pharmacy, Shahid Beheshti University of Medical Sciences, by voucher number of 1702.

A hundred gram of fresh aerial parts of the Dill was ground by the mixer and 40 mL of extract was prepared.

Matching to the extract volume (40 mL), sesame oil (from Saman Co.) was added to the mixture. Then the mixture was boiled with moderate heat for 2 h to evaporate its water content completely, leading to the oil phase. From this extraction, 44 g of the required product was gained.

*Animals*

Study was done on 18 male rats (*Rattus rattus*), with approximate weight ranges of 200 to 250 g. The rats’ paw volume were measured daily in the period of 8 days. The groups were as follows: Group 1 Formalin, Group 2 Dill Oil and Group 3 Diclofenac-gel.

Three groups of rats were randomly chosen with mere weight consideration (for the purpose of homogenizing the groups regarding their weights); sample volume was determined as 6 numbers per group with reference to the studies performed on rats regarding the inflammation (26-30).

*Plethysmometer*

The test process is as follows: the rat’s inflamed paw is put inside the liquid within the column to a specific area before and after the inflammation inducing and the weight is read. Then, the paw volume is easily calculated through the formula V = m/ρ (m is the mass of displaced fluid, equal to the shown weight; p is the fluid density and v is the volume of displaced fluid which equals to the entered paw volume). The difference between the paw volume before and after the inflammation is the edema volume.

*The test process*

On the first day, rats of each group were marked on their outer side of ankle; their paw volume were assessed from toe nails to the specified “marked” place by the new Plethysmometer, written in the relevant form, and immediately 0.05 mL formalin 2.5% was injected to the rats’ paw through a subcutaneous injection, using a conventional insulin syringe. The inflamed paw volume was measured after 1 h.

During the next days (day 2 to day 8) until the end of the study, the paw volume of each case was checked, documented and the drug was immediately rubbed topically on the inflamed area. The interval between the drug doses (24 h) and the temperature conditions of the site where the drug was kept were fixed as much as possible.

## Results and Discussion

Non-parametric Kruskal-Wallis test and Repeated Measures test were used for a comparison among groups regarding the treatment days and for checking the changes occurred in each group, respectively. ANOVA was used for a 2-by-2 comparison of groups in each day and Tukey was used for paired difference test comparisons.

*Group comparison regarding the change of Paw Volume*

The change of rats’ paw volumes were assessed among the three sample groups. Table 1 shows the change of rats’ paw volumes in the three groups from day 1 (before the injection) till day 8. As it can be seen, there has been no significant in the change of paw volume among the groups on day 1.

The average change of paw volume in the three groups after the inflammation induced with formalin proved to have no significant on day 1: formalin group: 0.31 (SEM = 0.02), Dill oil group: 0.30 (SEM = 0.01), Diclofenac group: 0.32 (SEM = 0.05).

**Table 1:** Average rats’ change of paw volume in three groups during the study

** Group**	**Formalin** **Mean ± SEM**	**Dill** **Mean ± SEM**	**Diclofenac Gel** **Mean ± SEM**
**Day**			
**1**st **Day (after inducing the inflammation)**	0.31 ± 0.022	0.30 ± 0.013	0.32 ± 0.052
**2**nd **Day**	0.44 ± 0.039	0.15 ± 0.047*	0.36 ± 0.086
**3**rd **Day**	0.34 ± 0.033	0.08 ± 0.026**	0.24 ± 0.069
**4**th **Day**	0.25 ± 0.032	0.06 ± 0.026**	0.16 ± 0.044
**5**th **Day**	0.22 ± 0.020	0.05 ± 0.025**	0.11 ± 0.040
**6**th **Day**	0.16 ± 0.025	0.04 ± 0.025*	0.11 ± 0.032
**7**th **Day**	0.10 ± 0.023	0.01 ± 0.014	0.06 ± 0.031
**8**th **Day**	0.12 ± 0.035	0.02 ± 0.017*	0.07 ± 0.030

*p-value less than 0.05 significant difference with formalin group.

**p-value less than 0.01 significant difference with formalin group.

It was clearly observed that from the 2nd day till the 6th day, the changes in the paw volume shifted to a quite significant, and based on the results, formalin group faced the most increase in the volume.

To explain more, on the 2nd day, taking into consideration the peak of inflammation, the results of the change of paw volume measurements among the three groups manifest that the average inflammations were as follows: formalin group: 0.44 (SEM = 0.03), Dill oil group: 0.15 (SEM = 0.04), Diclofenac group: 0.36 (SEM = 0.08) which had significant difference (p = 0.04). The third day showed a nearly similar pattern: formalin group: 0.34 (SEM = 0.03), Dill oil group: 0.08 (SEM = 0.02), Diclofenac group: 0.24 (SEM = 0.06), which had significant difference (p = 0.03).

In this experimental study, 3 groups (each group containing 6 rats) were compared during 8 days. On the first day after the injection of formalin and creation of paw inflammation in rats, there was no significant difference between these three groups. But comparing the paw volume changes in the 2nd to 6th days, there was significant difference between them (respectively, p-value: 0.01, 0.003, 0.007, 0.004 and 0.02). Statistical analysis showed that Dill group was the cause of this significant difference, therefore, paw volume changes in Dill group was less than other groups and the difference was significant.

**Figure 1:**
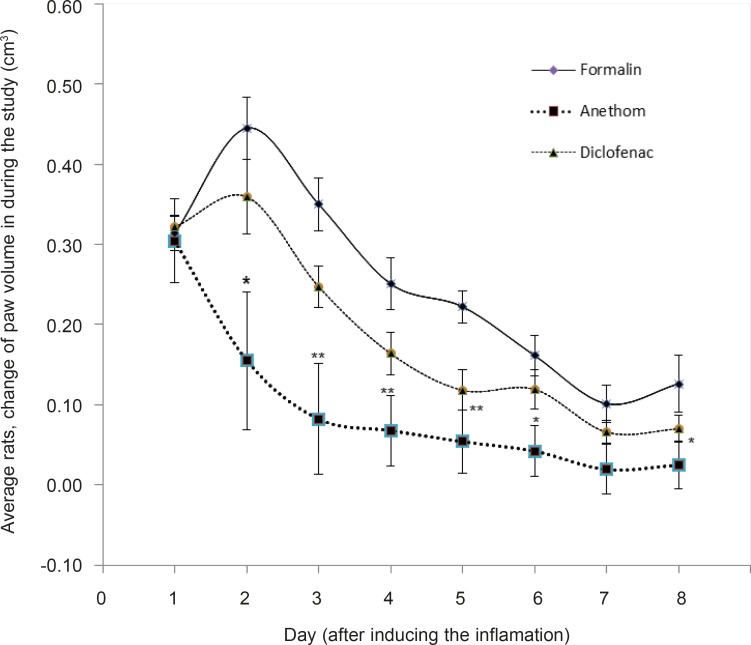
Average rats’ paw volume changes among the groups during the study. *p-value less than 0.05 considered a significant difference with formalin group. ** p-value less than 0.01 considered a significant difference with formalin group.

Comparing the statistics of paw volume changes from 2nd till 6th day, in which a significant difference was obvious, the analysis showed that the major source of the difference was the Dill oil; the rats’ paw volume of the Dill oil group was less than the other two groups whereas no significant difference was observed in the paw volumes changes of the other two groups, namely formalin and Diclofenac group (Figure 1).

In this experimental study, 3 groups (each group containing 6 rats) were compared during 8 days. On the first day after the injection of formalin and creation of paw inflammation in rats, there was no significant difference between these three groups. But comparing the paw volume changes in the 2nd to 6th days, there was significant difference between them (respectively, p-value: 0.01, 0.003, 0.007, 0.004 and 0.02). Statistical analysis showed that Dill group was the cause of this significant difference, therefore, paw volume changes in Dill group was less than other groups and the difference was significant.

## Discussion

Aromatic plants have been used as spice and medicine since the ancient Rome era (32). Spice herbs known in the ancient times are almost among the mint and Apiaceae family; some example are mint, oregano, basil, savory, parsley, Dill, and coriander (33).

A closer look at the anti-inflammatory effects of the different pharmaceutical forms of Diclofenac such as micro-emulsions and gels on the rat’s paw inflammation test shows the optimum effectiveness of the topical use of the drug forms (34).

The topical administration of Dill oil, prepared based on the Iranian Traditional Medicine, showed that this oil is capable of reducing the paw inflammation significantly (p < 0.001). It also determined that in Dill oil group, there has been a stronger reduction of inflammation compared to the Diclofenac group.

The results of the analysis performed on the composition of aerial parts and seed of Dill by the gas chromatography, show that 90% of the plant’s major components consists of d-carvone, d-limonene, and *α*-phellandrene and the other 10% includes components such as coumarin, flavonoids, fatty acids and protein (5, 23-25).

In some other studies, the anti-inflammatory and analgesic effects of carvone and limonene have been proved, as explained below. The hydro-alcoholic extract of the celery fruit has shown anti-inflammatory and analgesic effects (35). The celery extract contains limonene and selinene (36). Limonene has anti-inflammatory and analgesic effect (37). It controlled the cyclooxygenase 1 and 2 and inhibits the inflammation (38).

Carvone has shown a significant anti-inflammatory effect in the inflammatory carrageenan model (39). Based on the old sources of Iranian Traditional Medicine, oil is recommended as the base for getting the extracts out of aerial parts and seeds (6-13).

Chemical analysis of the Dill oil components through the gas chromatography has shown the existence of carvone and limonene. As the major contents of Dill oil (about 90%), the remarkable point in this study is the oil-based use extraction of Dill ingredients (40).

Another point worthy of notice is that the monoterpenic compounds such as carvone and limonene are insoluble in water. Therefore, the suitable solvents for their extraction are the oily and non-polar solvents (41).

These observations reveal that the traditional physicians have been quite careful on choosing the oil-based extract as a more effective form use of Dill. On the other hand, oil extract leads the non-polar compounds to be better absorbed; a fact that plays an important role in using this product (42).
